# Platelet reactivity in polymorbid older patients and the effect of 4-methylcatechol

**DOI:** 10.1186/s12877-026-07945-2

**Published:** 2026-07-17

**Authors:** Marcel Hrubša, Lukáš Konečný, Michaela Sirová, Slávka Homolková, Alejandro Carazo, Kateřina Matoušová, Lenka Kujovská Krčmová, Veronika Bernhauerová, Vladimír Blaha, Alena Šmahelová, Přemysl Mladěnka

**Affiliations:** 1 Department of Pharmacology and Toxicology, Faculty of Pharmacy in Hradec Králové, Charles University, Akademika Heyrovskeho 1203, Hradec Králové, 500 05 Czech Republic; 2 3rd Department of Internal Medicine-Metabolic Care and Gerontology, University Hospital and Faculty of Medicine in Hradec Králové, Charles University, Hradec Králové, Czech Republic; 3 Department of Clinical Biochemistry and Diagnostics, University Hospital Hradec Králové, Hradec Králové, Czech Republic; 4 Department of Analytical Chemistry, Faculty of Pharmacy in Hradec Králové, Charles University, Hradec Králové, Czech Republic; 5 Department of Biophysics and Physical Chemistry, Faculty of Pharmacy in Hradec Králové, Charles University, Hradec Králové, Czech Republic

**Keywords:** Antiplatelet drug, Older, Acetylsalicylic acid, Vorapaxar, Ticagrelor, Polyphenol

## Abstract

**Background:**

Older patients often suffer from multiple disorders and are hence frequently burdened by polypharmacy. Prevention of cardiovascular events using antiplatelet drugs might be indicated among older populations, but there are no studies assessing platelet function in these patients.

**Methods:**

Using impedance aggregometry, we compared platelet reactivity to 7 aggregation inducers between a group of 44 polymorbid older patients aged 78 + years (PP), and 50 generally healthy younger controls (median age, 44 years). None of the subjects were treated with antiplatelet therapy. We also examined their response to antiplatelet drugs (acetylsalicylic acid, ticagrelor, vorapaxar) and an experimental compound, 4-methylcatechol, a small polyphenol metabolite of many natural polyphenolic compounds. Data analysis was performed in the whole group as well as after removing or splitting the groups based on concomitantly administered drugs. Linear mixed-effects modelling was used to investigate the impact of both drugs and comorbidities on the obtained results.

**Results:**

A clinically achievable concentration of ASA (30 µM) failed to block platelet aggregation when arachidonic acid was used as an inducer, but the response to ticagrelor, vorapaxar, and 4-methylcatechol with relevant inducers was significant. However, the sensitivity to ticagrelor and vorapaxar was lower in PP when compared to healthy controls. The analysis also confirmed similar activity for 4-methylcatechol, but lower activity for acetylsalicylic acid in our PP. Further analyses excluding specific drugs did not significantly alter these outcomes. Inflammatory markers and the presence of type 2 diabetes mellitus were significant predictors of platelet reactivity while basic blood parameter data had no clear impact.

**Conclusions:**

The *ex vivo* response of platelets from PP to the standard antiplatelet drugs was lower. In contrast, they responded well to 4-methylcatechol.

**Supplementary Information:**

The online version contains supplementary material available at https://doi.org/10.1186/s12877-026-07945-2.

## Background

An increasingly aging population is a major concern for all developed countries as it creates a severe burden on the healthcare system, both in terms of inpatient and ambulatory care [1, 2]. While the impact of this trend may be ameliorated by implementing suitable health policies, specific considerations are required when designing pharmacotherapy for older populations. Due to the high prevalence of co-morbidities and age-related changes in pharmacokinetics and pharmacodynamics, there is an increased risk of side effects and drug-drug interactions [3, 4].

Cardiovascular disease is one of the leading causes of death, responsible for more than 30% of global mortality. Cardiovascular risk factors, such as hypertension, dyslipidaemia, diabetes, or overweight/obesity, are highly prevalent in older people; if left untreated, these conditions may lead to potentially life-threatening cardiovascular incidents, such as stroke or acute myocardial infarction [5].

Hence, the prevention of ischemic events by antiplatelet drugs is frequently indicated. Current antiplatelet therapy primarily utilizes acetylsalicylic acid (ASA) as an effective and inexpensive treatment. As an alternative, antagonists of the P2Y_12_ receptor for adenosine diphosphate (ADP) are used. In specific situations, such as acute coronary syndrome, dual antiplatelet therapy, consisting of a P2Y_12_ antagonist in combination with ASA is employed [6]. These drugs are not always suitable or effective as there is a high degree of resistance, contraindications, and a risk of drug-drug interactions. The latter two are particularly relevant in older populations due to the increased prevalence of many co-morbidities requiring a higher number of administered drugs [7, 8].

Additionally, due to a lack of studies focused specifically on older persons, who are often excluded from clinical studies owing to the presence of co-morbidities and polypharmacy, the current state of knowledge in antiplatelet treatment is very limited. This is highly concerning, as this is the part of the population that requires tailored and easy-to-follow, but effective pharmacotherapy. A novel antiplatelet drug with good efficacy that is also well tolerated could solve these problems and offer an alternative.

(Poly)phenolic metabolites of natural products may be a promising avenue. A polyphenol-rich diet has been associated with a significantly lower risk of the development and/or slower progression of cardiovascular disease in several epidemiological studies. As polyphenolic compounds generally have very low oral bioavailability, it was proposed that the actual compound(s) responsible for this effect are very likely intestinal metabolites of polyphenols formed by the gut microbiome [9, 10]. It was already shown that they have a much higher bioavailability, as well as a possible impact on the cardiovascular system by having antiplatelet and vasodilatory properties. One such metabolite is 4-methylcatechol (4-MC), which has already been demonstrated by our group to possess strong antiplatelet activity in healthy volunteers, patients with severe forms of familial hypercholesterolemic, and patients with type 1 diabetes mellitus. Furthermore, it demonstrated quantitatively stronger activity than ASA [11–13].

This study is hence a continuation of our previous research conducted on the aforementioned populations, which had 2 main aims: 1) to assess the effects of various platelet aggregation inducers and their respective inhibitors with clinical relevance in polymorbid older patients, and 2) to compare the effects of 4-MC and clinically used drugs ASA, ticagrelor and vorapaxar between these patients and a control group of generally healthy younger volunteers. In addition, a range of biochemical parameters along with currently administered drugs as well as medical histories were obtained and used for additional detailed analyses.

## Methods

### Agonists, inhibitors of platelet aggregation, and solvents

ASA, 9,11-dideoxy-11α,9α-epoxymethanoprostaglandin F_2α_ (U-46619), 4-MC, platelet-activating factor 16 (PAF), ristomycin monosulfate (ristocetin), and ticagrelor were purchased from Sigma-Aldrich (St. Louis, MO, USA), whereas vorapaxar was from Selleck Chemicals GmbH (Planegg, Germany). ADP, arachidonic acid, and thrombin receptor activating peptide 6 (TRAP) were purchased from Roche (Basel, Switzerland). Collagen was obtained from Diagnostica, a.s. (Prague, Czechia).

All antiplatelet compounds were dissolved in dimethyl sulphoxide (DMSO; Penta, Prague, Czechia). Ultrapure water was prepared using Milli-Q^®^ IQ 7000 (Merck Millipore, Massachusetts, USA), and saline (0.9% sodium chloride) was bought from B. Braun (Melsungen, Germany).

### Patient recruitment and blood collection

#### Inclusion criteria

Polymorbid older patients (PP) were included if they were aged 78 + years, hospitalized at the 3^rd^ Department of Internal Medicine-Metabolic Care, University Hospital in Hradec Králové, and diagnosed with three or more concurrent chronic health conditions. These conditions were generally of cardiovascular nature, such as stable hypertension, coronary heart disease, cerebrovascular, or peripheral artery diseases. They were also able to understand and sign an informed consent.

#### Exclusion criteria

Patients were excluded if they had diseases and conditions that are known to modify platelet function (e.g., acute infections and chronic inflammatory and autoimmune diseases, as well as malignancies), and/or interfere with drug pharmacokinetics (such as severe organ dysfunction – namely haematological, liver and renal impairment). Additional exclusion criteria included dementia of such severity that the patient was unable to understand and sign the informed consent (see Supplementary Table 1 for details), and terminally ill patients with symptomatic therapy.

Patients treated with antiplatelet drugs were generally excluded, apart from six patients administered a low dose of ASA. These six patients were included to demonstrate the differences in platelet responses and confirm the suitability of the method. The control group consisted of generally healthy younger subjects without any acute or unstable chronic disease, who undertook a preventive medical examination. 

Approximately 35 mL of blood was collected from every patient for our analysis. The specific types of tubes used, and corresponding volumes can be seen in Supplementary Table 2.

### Impedance aggregometry

To assess the *ex vivo* platelet activity and the antiplatelet effect of the compounds in our study, impedance aggregometry was utilised. This approach uses the aggregometer Multiplate (Roche, Rotkreuz, Switzerland) and allows for a wide range of both inducers and inhibitors of platelet aggregation. Experiments were always started precisely 30 min after the blood draw. Briefly, 300 μL of heparinized whole blood was diluted with 300 μL of saline in the Multiplate cuvettes, while being continuously stirred. Afterwards, each sample was incubated with either the tested compound or the solvent (DMSO, final concentration of up to 0.8%) for exactly 3 min. The incubation was followed by the addition of a chosen aggregation inducer, and the process was monitored for 6 min, yielding the final aggregometry data. Both incubation and measurement time conformed to the manufacturer’s instructions [14]. The concentrations of both the compounds and the inducers can be found in Supplementary Table 3.

### Biochemical analysis

Commonly measured metabolic markers (glucose, total cholesterol, HDL cholesterol, LDL cholesterol, and triglycerides) were measured in serum using standard commercial enzymatic kits employing the Cobas 8000 system (Roche, Basel, Switzerland). Creatinine and various inflammatory markers were determined in both serum and urine using a Prominence LC-20 HPLC system equipped with the SPD-M20A Shimadzu diode array detector (Shimadzu, Kyoto, Japan) with the stationary phase of two monolithic columns RP-18e (4.6 mm × 50 mm, 3.0 mm × 100 mm) connected together. A 15 mM phosphate buffer was employed as the mobile phase. Creatinine was detected at 235 nm using diode array detection [15].

### Statistical analysis

GraphPad Prism 11.0.0 (GraphPad Software, Boston, Massachusetts, USA) and R-studio (version 2026.04.0 + 526 "Globemaster Allium") [16] with packages from the tidyverse collection and corrplot were used for data manipulation, analysis, and visualization.

Normality of data was assessed using the Shapiro–Wilk test. For pairwise comparisons, the Mann–Whitney test or t-test was used. The family-wise error rate for multiple pairwise comparisons were corrected using the Benjamini–Hochberg method with a Q of 10%.

Numerical data are expressed as median with 95% confidence intervals. For multiple comparisons, the ordinary one-way ANOVA test followed by the Holm-Šídák multiple comparisons test or the Kruskal–Wallis test followed by the Dunn's multiple comparisons test were used. Categorical data were compared using the chi-square test.

For correlations, the non-parametric two-sided Spearman correlation coefficients were calculated. *A priori* power analyses were performed using G*Power 3.1.9.7 with an alpha level of 0.05 and a beta level of 0.95. Our sample size (44/50) allowed us to achieve an overall power of at least 0.95 with an effect size d = 0.8.

### Mathematical modelling—all data

Statistical significance was defined as a two-sided *p*-value < 0.05. All analyses were performed using the open-source statistics program JASP (version 0.19.3).

To evaluate the effects of age, clinical characteristics, and treatment type on platelet aggregation mean, a linear mixed-effects model was fitted. Platelet measurement was included as the dependent variable. Fixed effects included age, BMI, serum neopterin, treatment type, study group (geriatric *vs.* control), the treatment-by-group interaction, arterial hypertension, type 2 diabetes mellitus, dyslipidemia, chronic heart failure, and statins. A random intercept for patient identification number (ID) was included to account for repeated measurements obtained from the same individual under different treatment conditions. The model can be expressed as:

1 $$\begin{aligned}{Platelet }_{ij}=& {\beta}_{0}+{\beta}_{1}{\left(Treatment\right)}_{ij}+{\beta}_{2}{\left(Group\right)}_{i}\\&+{\beta}_{3}{(Treatment\times Group)}_{ij}+{\beta}_{4}{\left(Age\right)}_{i}\\&+{\beta}_{5}{\left(BMI\right)}_{i}+{\beta}_{6}{\left(Serum\,neopterin\right)}_{i}\\&+{\beta}_{7}{\left(Arterial\,hypertension\right)}_{i}\\&+{\beta}_{8}{\left(Type\,2\,Diabetes\right)}_{i}+{\beta}_{9}{\left(Dyslipidemia\right)}_{i}\\&+{\beta}_{10}{\left(Chronic\,heart failure\right)}_{i}\\&+{\beta}_{11}{\left(Anticoagulants-direct\,factor\,X\,inhibitors\right)}_{i}\\&+{\beta}_{12}{\left(Statins\right)}_{i}+{u}_{i}+{\epsilon}_{ij},\end{aligned}$$

where ${u}_{i}$ represents the patient-specific random effect and ${\varepsilon}_{ij}$ the residual error.

In the following analysis, 17 observations were removed by the software due to missing values. The random slopes for the factors Group, Age, BMI, Arterial hypertension, Type 2 diabetes and Dyslipidemia were removed by the software from the random effects grouping factor Patient ID because they did not vary within the levels of random effects of that grouping factor. The random slope of the factor Treatment was removed by the software from the random effects grouping factor Patient ID due to an insufficient number of observations. Thus, only the intercept was kept for random effects.

### Mathematical modelling—PP data (blood + liver markers)

Statistical significance was defined again as a two-sided *p*-value < 0.05, and all analyses were performed using JASP as well.

To evaluate the effects of blood, liver markers, and clinical characteristics on platelet aggregation mean in geriatric patients, a linear mixed-effects model was fitted. Platelet measurement was included as the dependent variable. Fixed effects included blood markers (hematocrit and platelet count), liver markers (alanine aminotransferase /ALT/ and alkaline phosphatase /ALP/), type 2 diabetes, and anticoagulants—direct factor X inhibitors.

A random intercept for patient identification number was included to account for repeated measurements obtained from the same individual under different treatment conditions. The model can be expressed as:

2 $$\begin{aligned}{Platelet }_{i}=& {\beta}_{0}+{\beta}_{1}{\left(Type\,2\,Diabetes\right)}_{i}\\&+{\beta}_{2}{\left(Anticoagulants-direct\,factor\,X\,inhibitors\right)}_{i}\\&+{\beta}_{3}{\left(Hematocrit\right)}_{i}\\&+{\beta}_{4}{\left(Platelet\,count\right)}_{i}+{\beta}_{5}{\left(ALT\right)}_{i}\\&+{\beta}_{6}{\left(ALP\right)}_{i}+{u}_{i}+{\epsilon}_{i},\end{aligned}$$

where ${u}_{i}$ represents the patient-specific random effect and ${\varepsilon}_{i}$ the residual error.

In the following analysis, 140 observations were removed by the software due to missing data points. The random slopes for all investigated factors were removed by the software from the random effects grouping factor Patient ID because they did not vary within the levels of random effects of that grouping factor. Thus, only intercept was kept for random effects.

## Results

A total of 50 PP were included in this study and compared to the data of the same number of generally healthy controls. Basic characteristics of both groups involved in the study are summarized in Table 1. The most common diagnoses are summarized in Table 2, and the pharmacotherapy is shown in Table 3. Liver function tests and basic blood panel were also recorded for PP (Supplementary Table 5–6). It was expected that there were significant differences between PP and controls, given the impossibility of recruiting fully healthy people from a similar age category (Supplementary Table 4). In addition, the PP group included more females and had only a single smoker. As for basic biochemical parameters, these patients had higher levels of serum glucose and triglycerides, and lower HDL cholesterol. In contrast, PP had better control of LDL, non-HDL, and total cholesterol levels. Creatinine levels differed significantly between older patients and the control group. As decreased kidney function leading to creatinine retention in older individuals is a known phenomenon, this difference was not surprising. Selected inflammatory markers were significantly higher in PP. Accordingly, there were significant differences in diagnoses and administered drugs (Tables 2, 3).

**Table 1:** Basic characteristics of polymorbid older patients and their comparison to the generally healthy control group

Group	Polymorbid older patients (*n* = 50)	Controls (*n* = 50)	***p* value**
Age (years)	83 (81—84)	44 (35—47)	**< 0.001**
Sex	13 M/37 F	27 M/23 F	**0.004**
BMI	27.19 (24.51–28.60)	26.19 (24.54–28.31)	0.98
Smoking (n)	1 (2%)	12 (24%)	**< 0.001**
Incidence of covid (n)^a^	17 (34.00%)	15 (30.00%)	0.67
Glucose (mM)	5.42 (5.16—5.95)	5.05 (4.80—5.30)	**0.02**
LDL (mM)	2.35 (2.03—2.75)	3.33 (3.09—3.52)	**< 0.001**
HDL (mM)	1.09 (0.980—1.18)	1.45 (1.33—1.66)	**< 0.001**
Triglycerides (mM)	1.43 (1.22—1.62)	1.21 (1.06—1.38)	**0.03**
non-HDL cholesterol (mM)	3.15 (2.52—3.44)	3.89 (3.64—4.01)	**< 0.001**
Total cholesterol (mM)	4.04 (3.78—4.42)	5.39 (5.05—5.69)	**< 0.001**
Serum creatinine (µM)	99.14 (91.68–115.5)	74.52 (69.32–82.12)	**< 0.001**
Serum neopterin (nM)	15.69 (12.84–21.67)	7.13 (6.52–7.79)	**< 0.001**
Serum tryptophan (µM)	38.3 (29.95–43.68)	66.2 (61.85–70.07)	**< 0.001**
Serum kynurenine (µM)	3.03 (2.55–3.64)	2.17 (2.09–2.31)	**< 0.001**
Serum kynurenine/tryptophan ratio	89.26 (62.93–106.6)	32.91 (31.48–36.96)	**< 0.001**
Urine kynurenine/creatinine ratio	1.08 (0.63–1.79)	0.22 (0.19–0.28)	**< 0.001**
Urine neopterin/creatinine ratio	380 (306–431)	164 (152–185)	**< 0.001**
Urine tryptophan/creatinine ratio	3.94 (3–4.8)	4.4 (3.8–5.3)	**< 0.001**
Urine kynurenine/tryptophan ratio	280.3 (185–370.5)	55.69 (49.73–59.04)	**< 0.001**

Data expressed as median with 95% confidence intervals. Normality was assessed using the Shapiro–Wilk test. Statistical significance for continuous variables was tested by the t-test or the Mann–Whitney test for non-parametric variables. Chi-square test was used in the case of categorical variables. Significant differences are emphasized by bold numbers

*BMI* Body mass index, *LDL* Low-density lipoprotein, *HDL* High-density lipoprotein

^a^diagnosed from the start of the COVID pandemic until the period before donor recruitment, however, no acute infection patients were included in the study

**Table 2:** The most common diagnoses of polymorbid older patients

Diagnosis	Polymorbid older patients	Controls	*p* value
Arterial Hypertension	40	10	< 0.001
Allergy	2	10	0.01
Dyslipidemia	23	0	< 0.001
Chronic Heart Failure	17	0	< 0.001
Atrial Fibrillation	16	0	< 0.001
Structural Heart Defects*	12	0	< 0.001
Type 2 Diabetes mellitus	10	0	< 0.001

Statistical significance was assessed by the Chi-square test

*Heart valve defects and/or insufficiencies, atrial and/or ventricular dilation

**Table 3:** Drugs administered to polymorbid older patients (PP) in this study and comparison to generally healthy population

Drug group ATC	Drug group name	C	PP	*p* value
A02BC	Proton pump inhibitors	3	31	**< 0.001**
B01AB	Anticoagulants—heparins	0	25	**< 0.001**
C03CA01	Loop diuretics (solely furosemide)	0	25	**< 0.001**
C07AB	Beta-blockers (all cardioselective)	0	22	**< 0.001**
N02BB02	Metamizol and combination (pitofenone)	0	22	**< 0.001**
C09A + B	ACE inhibitors and combination	0	18	**< 0.001**
B01AF	Anticoagulants—direct factor X inhibitors	0	17	**< 0.001**
C10AA	Hypolipidemics—HMG-CoA reductase inhibitors (statins)	0	16	**< 0.001**
J01C	Penicillins^a^	0	16	**< 0.001**
R03A	Sympathomimetics and combinations (inhalators)	5	15	**0.01**
C08	Dihydropyridines and combinations	5	15	**0.01**
A12BA01	Potassium chloride	0	14	**< 0.001**
N05BA	Benzodiazepines	0	14	**< 0.001**
N06A	Antidepressants	0	12	**< 0.001**
A06AD11	Lactulose	0	10	**0.001**
M04AA01	Allopurinol	1	10	**0.004**
H03AA01	Thyroid hormones (solely levothyroxine)	6	10	0.28
R05CB	Mucolytics	0	9	**0.002**
N02BF	Gabapentinoids	0	9	**0.002**
	Probiotics	0	9	**0.002**
C09C/D	Sartans and combinations	9	8	0.79
A10A	Insulins	0	7	**0.006**
A12AX	Calcium and its combination	0	7	**0.006**
B01AC06	Acetylsalicylic acid	0	6	**0.01**
C03DA01	Spironolactone	0	7	**0.006**
H02AB	Glucocorticoids-systemic	0	6	**0.01**
N02BE01	Paracetamol	0	6	**0.01**
R06A	Antihistamines	8	3	**0.11**
C02AC	Imidazoline antagonists	0	6	**0.01**
C05CA	Flavonoids^b^	2	6	**0.14**
J01FA	Macrolides (solely clarithromycin)	0	6	**0.01**
G04	Urologics (parasympatholytics or α_1A_-antagonists) ^c^	0	6	**0.01**
J01DD	Cephalosporins	0	5	**0.02**
B03BB	Folic acid and derivatives	0	5	**0.02**
N02A	Analgesics—opioids	0	5	**0.02**
R03B	Inhaled Glucocorticoids	0	2	0.15

Statistical significance was assessed by the Chi-square test

Only PP patients that were not treated with acetylsalicylic acid were included (*n* = 44)

*ACE* Angiotensin converting enzyme, *C* Control

^a^and their combinations with beta lactamase inhibitors; ^b^only purified flavonoid micronized fraction; ^c^ solifenacin, darifenacin and tamsulosin

Significant differences are emphasized by bold numbers

As a first step, the *ex vivo* effect of clinically used drugs and the active phenolic metabolite 4-MC was tested in all PP. Since the use of antiplatelet drugs can distort the results, all 6 patients treated with ASA were removed, and hence only 44 PP were analysed. As expected, these six patients reacted differently to arachidonic acid and collagen compared to the rest of the patients, while retaining the same response to other inducers and drugs, which was in line with the mechanism of action of ASA (Supplementary Data Figure S1).

**Fig. 1:**
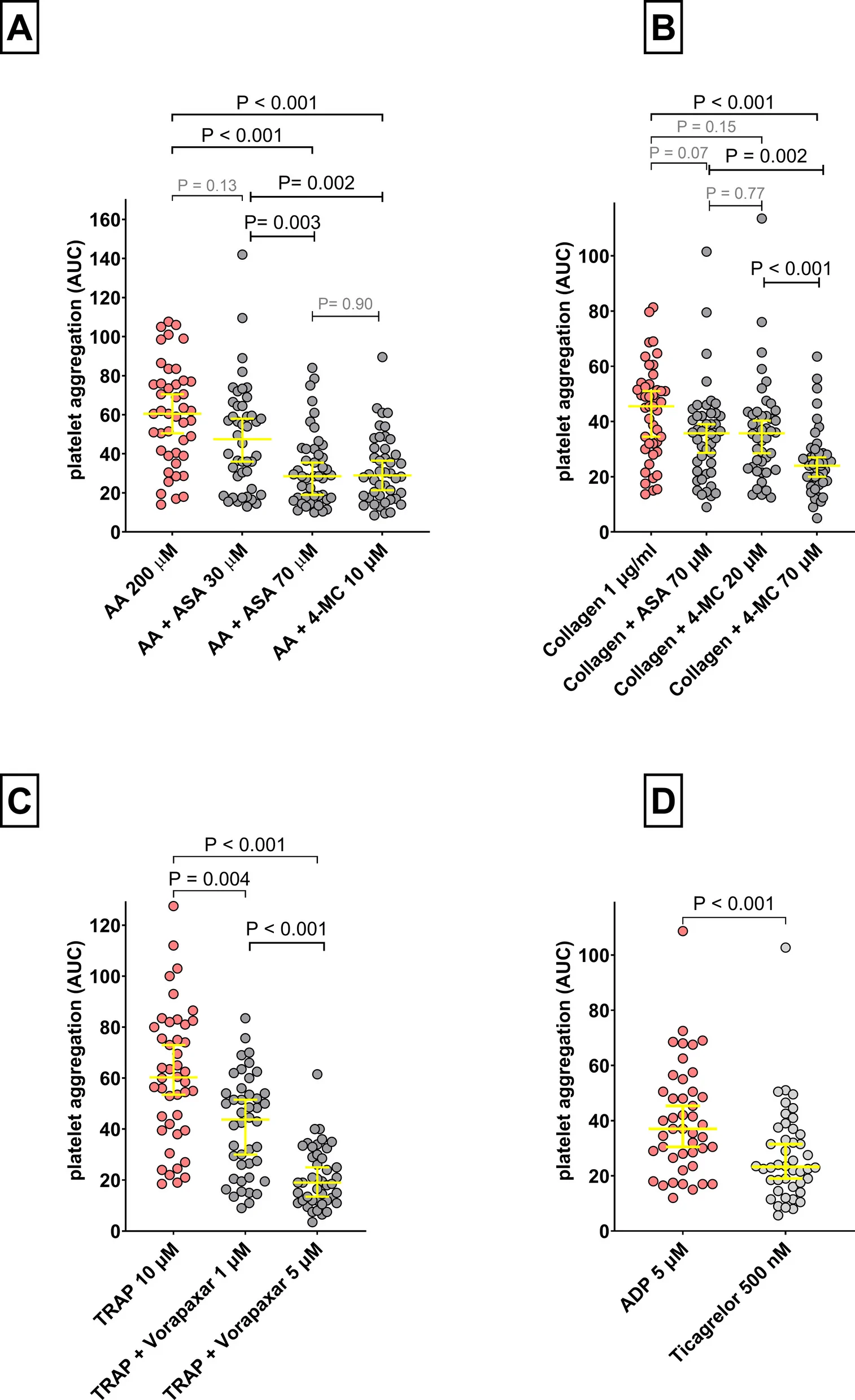
The antiplatelet effect of the tested drugs in polymorbid older patients. DMSO was used as a control for each inducer, the concentration of inducer used is shown in the first column. Non-normal distribution was found in all cases through the Shapiro–Wilk test. Statistical significance was calculated using the non-parametric Kruskal–Wallis test followed by the post hoc Dunn's multiple comparisons test. Pairwise comparisons were performed with the non-parametric Mann–Whitney test. (**A**) arachidonic acid (AA), (**B**) collagen, (**C**) thrombin receptor agonist peptide 6 (TRAP), (**D**) adenosine diphosphate (ADP). ASA = acetylsalicylic acid, 4-MC = 4-methylcatechol

When we analysed the absolute values (measured as area under the curve /AUC/ of platelet aggregation) in the tested population, ASA in a concentration of 30 µM, which is a concentration roughly achievable with a low-dose ASA therapy, was ineffective in arachidonic acid-triggered platelet aggregation. Only its higher concentration of 70 µM, unachievable in plasma from a low-dose ASA regimen, was active. By contrast, 4-MC was active at a concentration of 10 µM, and the effect was similar to the higher concentration of ASA (Fig. 1A). Concerning collagen, ASA in the above-mentioned higher concentration of 70 µM was numerically but not significantly active (*p* = 0.07). A low concentration of 4-MC (20 µM) was also inactive (*p* = 0.15) while the higher concentration (70 µM) was active (*P* < 0.001, Fig. 1B). In the case of TRAP, concentration-dependent antiplatelet effects of the thrombin receptor PAR-1 antagonist, vorapaxar, were observed (Fig. 1C). Ticagrelor, a P2Y_12_ antagonist, was also clearly active when compared to the corresponding negative control (Fig. 1D).

The individual relative responses in percent were also analysed. They were calculated as the inhibition of platelet aggregation for each donor related to the samples treated solely with the solvent and the corresponding inducer (Supplementary Data Figure S2). The results corresponded to the absolute value outcomes.

**Fig. 2:**
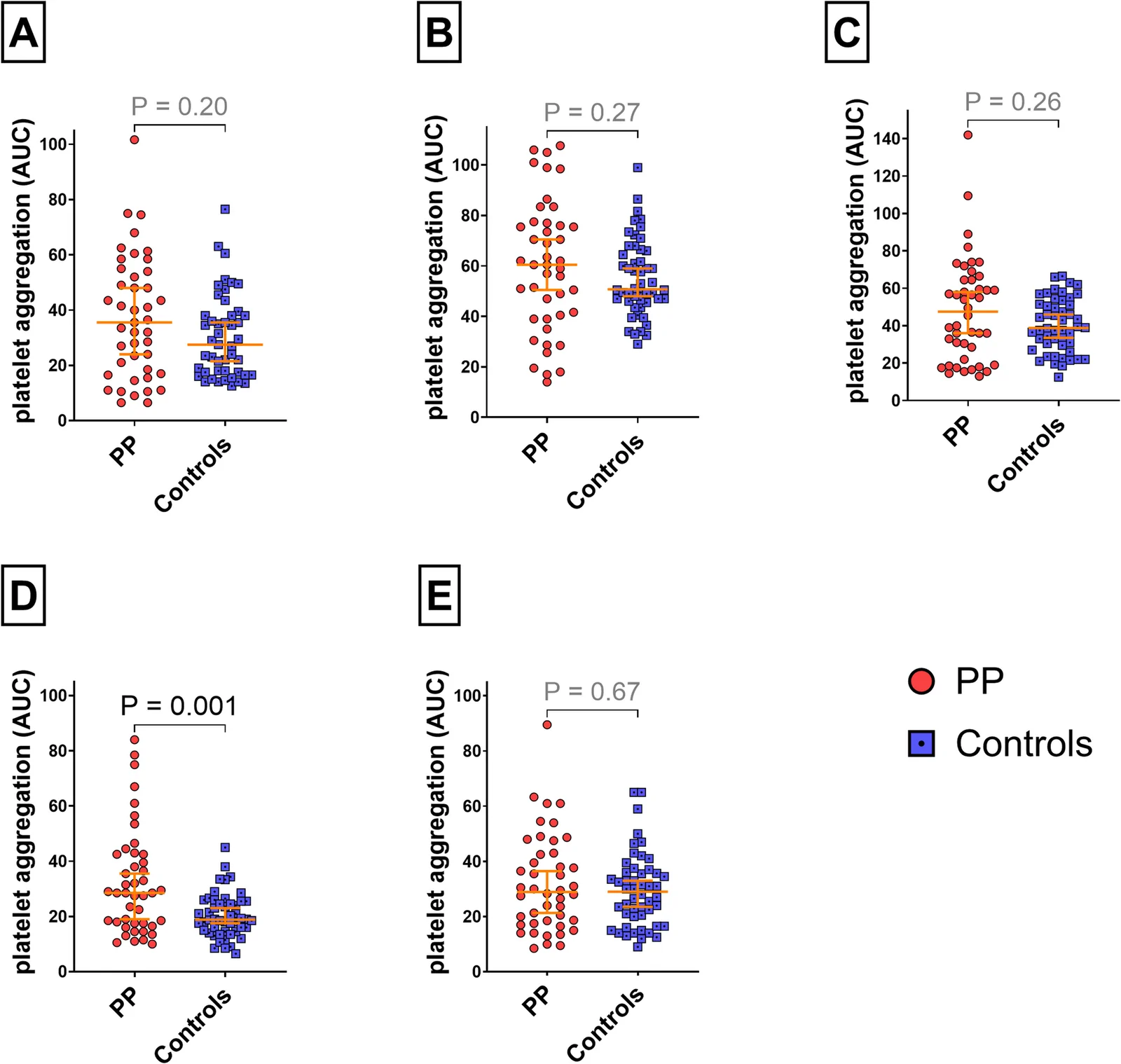
Pairwise comparison of responses to arachidonic acid and its inhibition in polymorbid older pa-tients (PP, red) and healthy controls (blue). Data expressed as the area under the curve (AUC). Normality was assessed using the Shapiro-Wilk test, and statistical significance was calculated using the Mann-Whitney test or the unpaired t-test, respectively. (**A**) arachidonic acid (AA) – low concentration (60 µM), (**B**) AA – high concentration (200 µM), (**C**) AA 200 µM after pretreatment with acetylsalicylic acid 30 µM, (**D**) AA 200 µM after pretreatment with acetylsalicylic acid (70 µM), (**E**) AA 200 µM after pretreatment with 4-methylcatechol (10 µM)

In the second step, the impact of platelet aggregation inducers and antiplatelet compounds was compared between the tested group of PP and generally healthy donors. There were no significant differences in AUC responses to either concentration of arachidonic acid (Fig. 2AB). Both groups also reacted similarly to 4-MC and the lower concentration of ASA, while the effect of ASA at a concentration of 70 µM was significantly lower in PP (Fig. 2CDE). Even when the data were analysed as relative values (Supplementary Figure S4), i.e., per cent response *vs.* the effect of arachidonic acid in each individual blood donor, the same outcomes were achieved, meaning ASA at the higher concentration was clearly less active in PP.

**Fig. 3:**
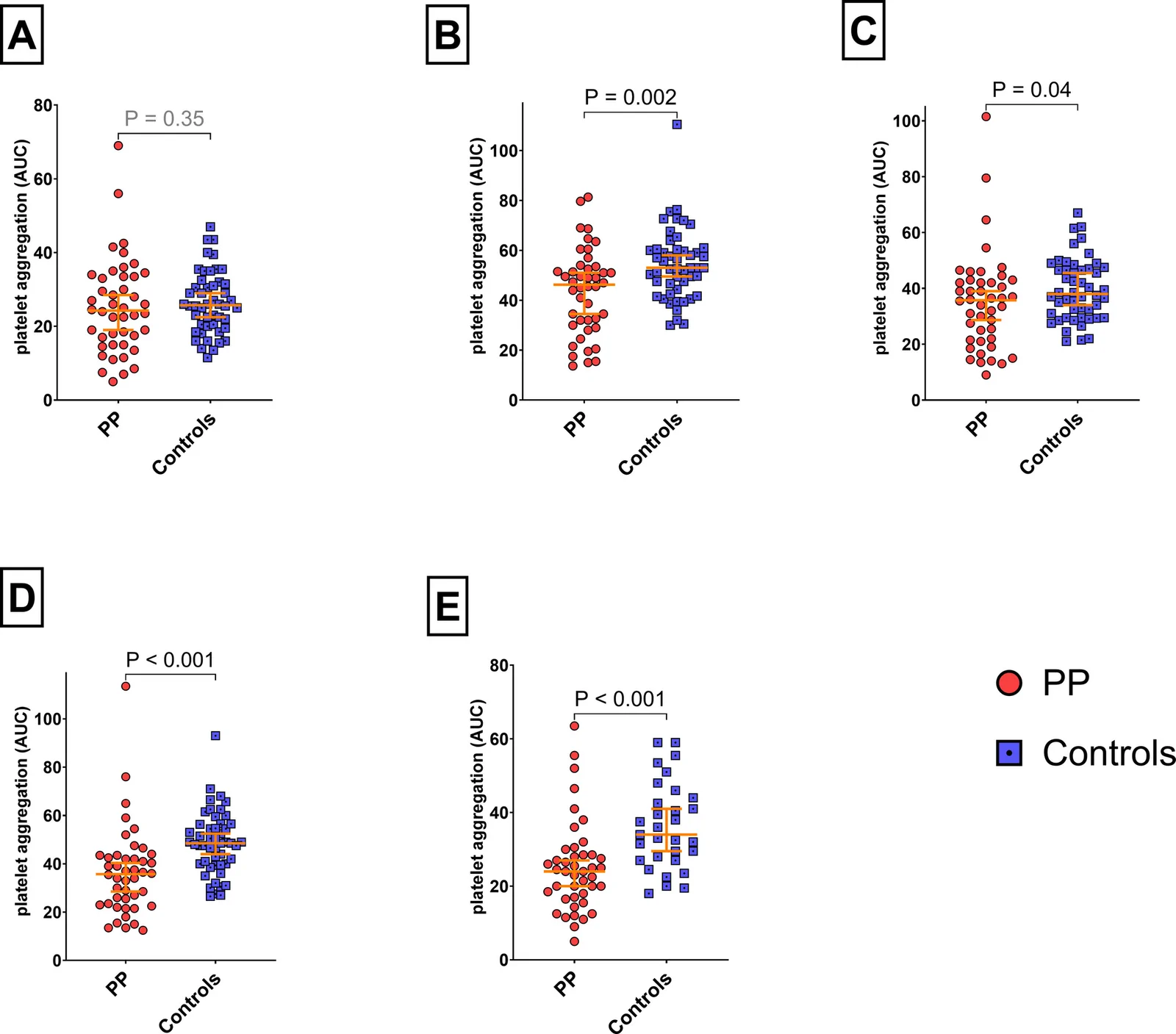
Pairwise comparison of responses to collagen and its inhibition in polymorbid older patients (PP, red) and generally healthy controls (blue). Data expressed as the area under the curve (AUC). Normality was assessed using the Shapiro–Wilk test and statistical significance was calculated using the Mann–Whitney test/unpaired t-test. (**A**) Collagen in a low concentration of 0.16 µg/mL, (**B**) Collagen in a higher concentration of 1 µg/mL, (**C**) Collagen 1 µg/mL after pretreatment with acetylsalicylic acid (70 µM), (**D**) Collagen 1 µg/mL after pretreatment with 4-methylcatechol (20 µM), (**E**) Collagen (1 µg/mL) after pretreatment with 4-methylcatechol (70 µM)

Unexpectedly, there was a significantly lower response to the 1 μg/mL dose of collagen in PP (Fig. 3B). Accordingly, the AUC values for both ASA and 4-MC were lower in these patients when absolute values were analysed (Fig. 3CDE). Interestingly, when individual relative per cent responses were analysed, 4-MC at a lower concentration was still more active compared to ASA, whose effect did not differ between the generally healthy and PP samples (Supplementary Figure S4).

**Fig. 4:**
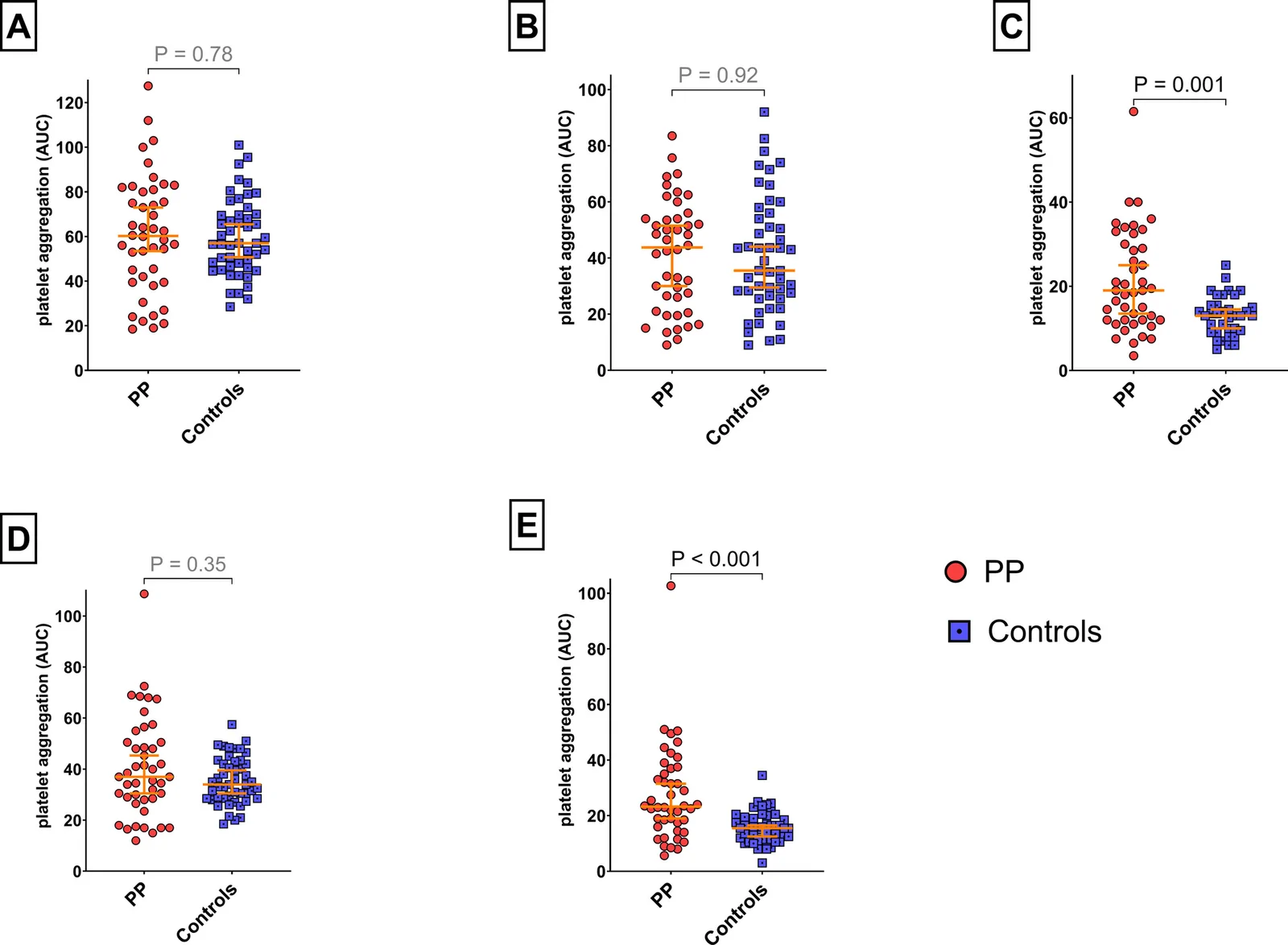
Pairwise comparison of responses to adenosine diphosphate and thrombin receptor agonist peptide 6 (TRAP) and its inhibition in polymorbid older patients (PP, red) and generally healthy controls (blue). Data expressed as the area under the curve (AUC). Normality was assessed using the Shapiro–Wilk test. Statistical significance was calculated using the Mann–Whitney test or the unpaired t-test. (**A**) TRAP (10 µM), (**B**) TRAP (10 µM) after pretreatment with vorapaxar (1 µM), (**C**) TRAP (10 µM) after pretreatment with vorapaxar (5 µM), (**D**) ADP (5 µM) (**E**) ADP (5 µM) after pretreatment with ticagrelor (500 nM)

TRAP, a thrombin receptor agonist, induced the same degree of platelet aggregation in both groups, while a thrombin PAR1 antagonist, vorapaxar, at a higher concentration of 5 µM was more potent in the control group (Fig. 4AC). However, its lower concentration was equally potent in both groups of blood donors (Fig. 4B). The relative percent analysis yielded the same results (Supplementary Data Figure S4).

Platelet aggregation induced by ADP was similar in generally healthy persons and PP, but the ADP receptor P2Y_12_ antagonist ticagrelor was much more potent in controls than in patients (Fig. 4DE). The same effect was observed in individual relative per cent change analysis (Supplementary Data S4).

**Fig. 5:**
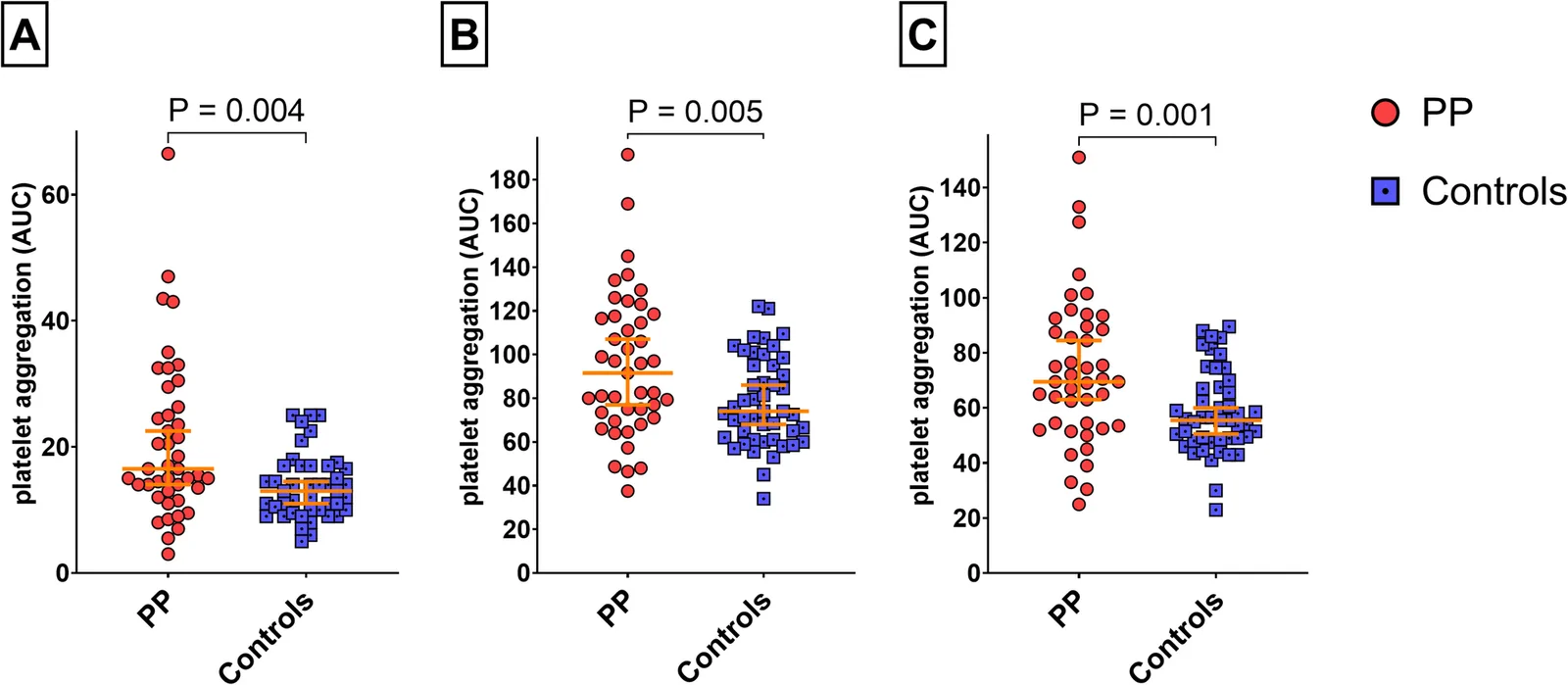
Pairwise comparison of responses to ristocetin and its inhibition in polymorbid older patients (PP, red) and healthy controls (blue). Data expressed as the area under the curve (AUC). Normality was assessed using the Shapiro–Wilk test. Statistical significance was calculated using the Mann–Whitney test/unpaired t-test. (**A**) ristocetin in a low concentration (4 µM), (**B**) ristocetin in a high concentration (400 µM), (**C**) ristocetin (400 µM) after pretreatment with 4-methylcatechol (240 µM)

The response to ristocetin, both in low and high concentrations, was significantly higher in PP. In contrast to collagen, the effect of 4-MC (tested in a very high concentration solely for experimental reasons) on ristocetin-induced aggregation was also significantly lower in healthy controls (Fig. 5). Due to the overall lower response to ristocetin in healthy patients, there were, however, no significant differences in terms of percent of response to 4-MC (Supplementary Data Figure S4). There were also no differences in platelet responses to either U-46619 or PAF between groups (Supplementary Data Figure S5).

As PP were frequently treated with many drugs, further analyses were performed to detect potential confounders. In the case of a high number of patients receiving one category of drugs, the whole group was divided into two based on the administration of a particular drug or its absence (Table 4). In cases of a smaller number of patients taking systemic glucocorticoids and flavonoids, both of which may be relevant for platelet reactivity, the analyses were re-performed excluding these patients (Table 5). In the majority of cases, solely the degree of significance but not significance itself changed.

**Table 4:** Pairwise comparison in polymorbid older patients taking specific drug group *vs.* the remaining patients

	Proton pump inhibitors	Heparins	Furosemide	Beta-blockers	Metamizol and combinations
Arachidonic acid 60 µM	0.43	0.89	0.28	0.90	0.71
Arachidonic acid 200 µM	0.79	0.47	0.63	0.90	0.80
Arachidonic acid 200 µM + ASA 30 µM	0.03*	0.99	0.33	0.23	0.30
Arachidonic acid 200 µM + ASA 70 µM	0.11	0.60	0.99	0.84	0.26
Arachidonic acid 200 µM + 4-MC 10 µM	0.69	0.84	0.34	0.86	0.43
Collagen 0.16 µg/mL	0.95	0.35	0.25	0.83	0.55
Collagen 1 µg/mL	0.47	0.30	0.86	0.23	0.86
Collagen 1 µg/mL + ASA 70 µM	0.95	0.07	0.33	0.52	0.69
Collagen 1 µg/mL + 4-MC 20 µM	0.96	0.49	0.54	0.43	0.42
Collagen 1 µg/mL + 4-MC 70 µM	0.48	0.06	0.58	0.36	0.22
ADP 5 µM	0.90	0.24	0.55	0.93	0.76
ADP 5 µM + Ticagrelor 500 nM	0.50	0.48	0.41	0.62	0.97
TRAP 10 µM	0.19	0.47	0.60	0.51	0.65
TRAP 10 µM + Vorapaxar 1 µM	0.86	0.70	0.42	0.76	0.56
TRAP 10 µM + Vorapaxar 5 µM	0.85	0.19	0.72	0.11	0.34
Ristocetin 4 µM	0.79	0.48	0.48	0.10	0.42
Ristocetin 400 µM	0.87	0.32	0.54	0.88	0.77
U-46619	0.87	**0.009** ^#^	0.96	0.78	0.93
PAF 20 nM	0.93	0.11	0.54	0.69	0.65

All patients with ASA were excluded from the analysis. Normality was assessed using the Shapiro–Wilk test. Statistical significance was calculated using the Mann–Whitney test/unpaired t-test. Numbers represent *p*-values. Significant differerences are emphasized by bold numbers

*ADP* Adenosine diphosphate, *ASA* Acetylsalicylic acid, *4-MC* 4-methylcatechol, *PAF* Platelet-activating factor-16, *TRAP* Thrombin receptor agonist peptide-6

^*^higher AUC/lower % of inhibition in drug group

^#^higher AUC/lower % of inhibition in non-drug group

**Table 5:** Pairwise comparison of the platelet aggregation between polymorbid older patients and generally healthy controls after removal of blood donor being administered with glucocorticoids and flavonoids

	p values		
	no ASA	no glucocorticoids	no flavonoids
Arachidonic acid			
Arachidonic acid 60 µM	0.2	0.15	0.29
Arachidonic acid 200 µM	0.27	0.45	0.21
Arachidonic acid 200 µM + ASA 30 µM	0.26	0.24	0.10
Arachidonic acid 200 µM + ASA 70 µM	**0.001**	**0.001**	**0.002**
Arachidonic acid 200 µM + 4-MC 10 µM	0.67	0.63	0.74
Arachidonic acid 200 µM + ASA 30 µM %	0.12	**0.04**^**a**^	0.21
Arachidonic acid 200 µM + ASA 70 µM %	** < 0.001**	** < 0.001**	** < 0.001**
Arachidonic acid 200 µM + 4-MC 10 µM	0.6	0.37	0.72
Collagen			
Collagen 0.16 µg/mL	0.35	0.57	0.40
Collagen 1 µg/mL	**0.002**	**0.003**	**0.005**
Collagen 1 µg/mL + ASA 70 µM	**0.04**	0.07	0.06
Collagen 1 µg/mL + 4-MC 20 µM	** < 0.001**	** < 0.001**	** < 0.001**
Collagen 1 µg/mL + 4-MC 70 µM	** < 0.001**	** < 0.001**	** < 0.001**
Collagen 1 µg/mL + ASA 70 µM %	0.28	0.28	0.40
Collagen 1 µg/mL + 4-MC 20 µM %	** < 0.001**	** < 0.001**	** < 0.001**
Collagen 1 µg/mL + 4-MC 70 µM %	0.25	0.38	0.11
Ristocetin			
Ristocetin 4 µM	**0.004**	**0.002**	**0.001**
Ristocetin 400 µM	**0.005**	**0.02**^**a**^	**0.002**
Ristocetin 400 µM + 4-MC 250 µM	**0.001**	**0.006**	** < 0.001**
Ristocetin 400 µM + 4-MC 250 µM %	0.72	0.69	0.56
TRAP			
TRAP 10 µM	0.78	0.81	0.68
TRAP 10 µM + Vorapaxar 1 µM	0.92	0.96	> 0.99
TRAP 10 µM + Vorapaxar 5 µM	**0.001**	** < 0.001**	**0.004**
TRAP 10 µM + Vorapaxar 1 µM %	0.71	0.60	0.57
TRAP 10 µM + Vorapaxar 5 µM %	** < 0.001**	** < 0.001**	** < 0.001**
Adenosine diphosphate (ADP)			
ADP 5 µM	0.35	0.28	0.20
ADP 5 µM + Ticagrelor 500 nM	** < 0.001**	** < 0.001**	** < 0.001**
ADP 5 µM + Ticagrelor 500 nM %	** < 0.001**	** < 0.001**	** < 0.001**
Other inducers			
U-46619	0.80	0.67	0.80
PAF 20 nM	0.75	0.74	0.71

Normality was assessed using the Shapiro Wilk test. Statistical significance was calculated using the Mann–Whitney test or unpaired t-test Numbers represent *p*-values. Significant differerences are emphasized by bold numbers

*ADP* Adenosine diphosphate, *ASA* Acetylsalicylic acid, *4-MC* 4-methylcatechol, *PAF* Platelet-activating factor-16, *TRAP* Thrombin receptor agonist peptide 6

^a^lower effect in older patients (higher AUC/lower % of inhibition); ASA = acetylsalicylic acid

In only four instances did these drugs significantly affected the response to platelet inducers and/or antiplatelet drugs (Tables 4 and 5): 1) PP treated with proton pump inhibitors responded less to ASA at a concentration of 30 µM. 2) Patients administered heparin and/or its analogues showed a lower response to the thromboxane receptor agonist, U-46619. 3) The removal of glucocorticoids resulted in a significant difference in the response to the lower concentration of ASA in the AA-induced aggregation (*p* = 0.04) as it became significantly lower in the PP (Supplementary Figure S6), and 4) conversely, the removal of either glucocorticoids or flavonoids led to the disappearance of significant differences in the response to collagen after pretreatment with 70 µM ASA (*p* = 0.07 or 0.06, respectively). In the case of multiple comparisons, *p*-values were adjusted using the Benjamini–Hochberg method (Supplementary Table 7–8). These corrections showed that the effect of ASA 70 µM was not significant in collagen-induced aggregation. Also, the effect of proton pump inhibitors was not significant in drug split analyses (ASA, 30 µM), similarly to the effect of glucocorticoid removal (also ASA, 30 µM).

**Fig. 6:**
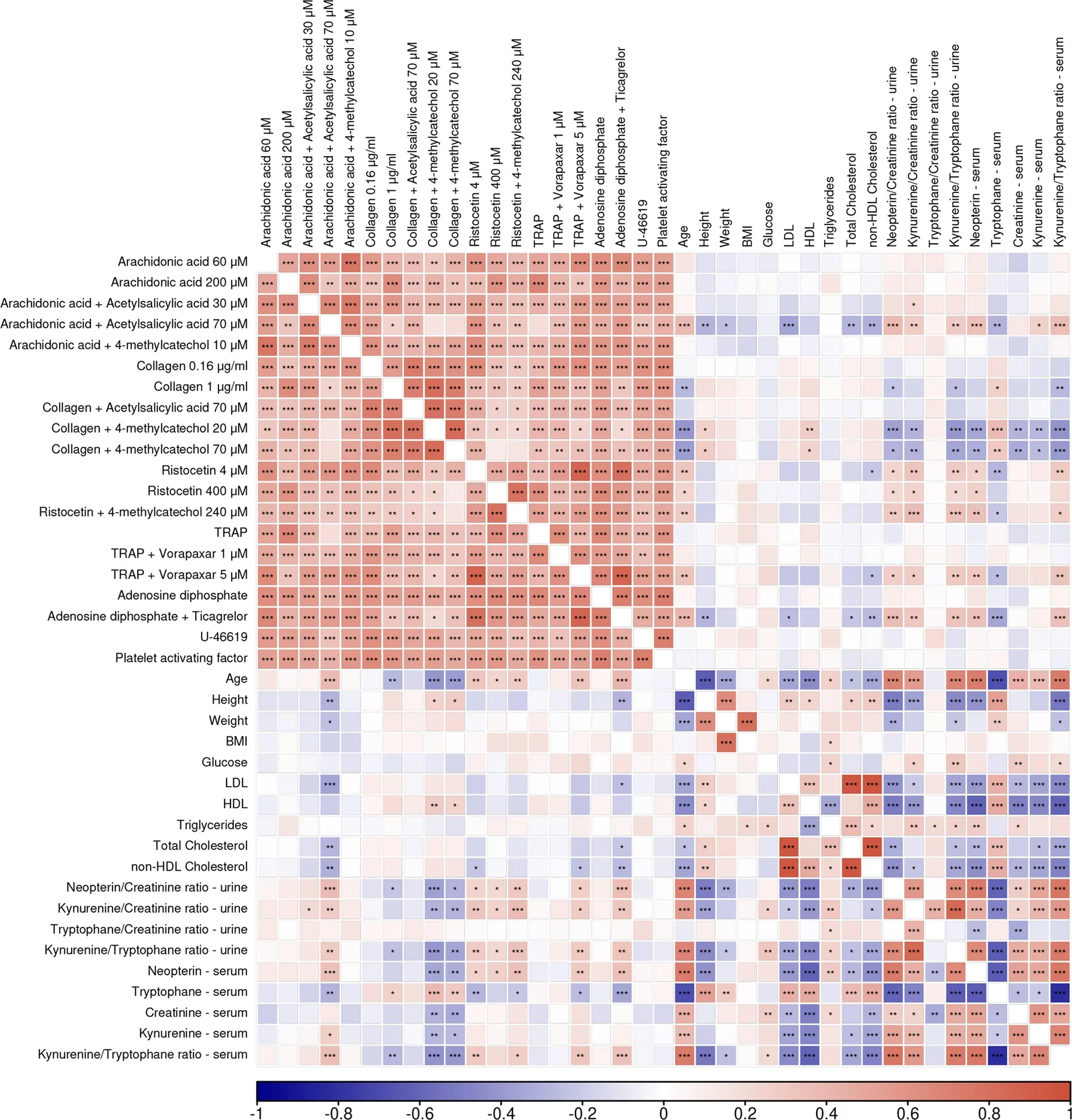
Correlation analysis of anthropometric, biochemical, and aggregometry values. Normality was assessed using the Shapiro–Wilk test. The majority of variables were non-parametric, therefore two-sided Spearman correlation coefficients were calculated. The entire control group and polymorbid older patients without acetylsalicylic acid were included in the analysis

Our analyses of various anthropometric and biochemical parameters have shown several significant correlations (Fig. 6, Supplementary Data Figures S7-10). Their significances were, however, rather low in most cases, barring the obvious exceptions, such as connected pathways and co-dependent variables.

Next, a linear mixed-effects model was fitted to evaluate the effects of age, clinical characteristics and treatment type on platelet aggregation data. As LDL, HDL, triglycerides, non-HDL cholesterol, and total cholesterol were significantly correlated (for all combinations, the *p*-value of Pearson’s coefficient was < 0.001), dyslipidemia was selected as a common characteristic. Similarly, serum neopterin, serum tryptophane, serum creatinine, and serum kynurenine were significantly correlated (for all combinations, the *p*-value of Pearson’s coefficient was < 0.001). Thus, only serum neopterin was selected as a common characteristic.

Serum neopterin (i.e., inflammatory parameters), type 2 diabetes, and type of treatment-by-group interaction (indicating that differences in platelet aggregation response between control and elderly subjects varied according to treatment) were significant predictors of the degree of platelet aggregation, while group (control or PP) as an individual factor was not a significant predictor of platelet aggregation (Table 6).

**Table 6:** Factors influencing platelet aggregation predicted by the linear mixed-effects model

Effect	*p*-value
age	0.607
BMI	0.232
neopterin—serum	**0.014**
arterial hypertension	0.942
dyslipidemia	0.539
chronic heart failure	0.381
type 2 diabetes mellitus	**0.005**
anticoagulants—direct factor X inhibitors	0.091
statins	0.888
group	0.886
treatment	** < 0.001**
group × treatment	** < 0.001**

*BMI* Body mass index

Significant differerences are emphasized by bold numbers

As an alternative, we fitted a linear mixed-effects model in which we removed dyslipidemia as a factor and included the factors LDL, HDL, and triglycerides. None of these factors was determined to be a significant predictor of platelet aggregation.

The analysis determined that serum neopterin was positively associated (slope = 0.242) with platelet aggregation. Post-hoc analysis of estimated marginal means for the factor Type 2 diabetes mellitus revealed that the patients without type 2 diabetes had lower average platelet aggregation than the patients with type 2 diabetes by 14.16 units (*p*-value = 0.004, adjusted using Holm correction).

In the last step, the possible impact of liver function and blood cell count was assessed in PP patients in a linear mixed-effects model. Concentrations of white blood cells correlated with the concentrations of erythrocytes, platelets, and hematocrit. As expected, red blood cell count correlated with hematocrit (*p* < 0.001); therefore, hematocrit and platelets were selected as common characteristics. A similar situation was noted with serum enzymes. Serum ALT was correlated with both serum aspartate aminotransferase and gamma-glutamyl transferase (*p* < 0.001). Aspartate aminotransferase was correlated with gamma-glutamyl transferase (*p* < 0.001) and serum ALP was correlated with gamma-glutamyl transferase (*p* < 0.001), only ALT and ALP were selected as common characteristics. None of the selected liver or blood count parameters predicted platelet aggregation, while type 2 diabetes was still a significant factor even in this PP analysis (Table 7).

**Table 7:** Factors influencing platelet aggregation predicted by the linear mixed effects model

Effect	*p*
serum alanine aminotransferase (µcat/L)	0.547
Hematocrit	0.388
Platelets (10^9^/L)	0.252
serum alkaline phosphatase (µcat/L)	0.706
Type 2 Diabetes	**0.026**
Anticoagulants—direct factor X inhibitors	0.195

Significant differences are emphasized by bold numbers

## Discussion

Pharmacotherapy of older persons clearly requires a unique approach, mainly due to the increased risk of side effects, frequent polypharmacy, and the presence of various comorbidities [17]. There are currently no available studies examining platelet reactivity specifically in older patients suffering from several comorbidities. There are also no studies examining the effect of commonly used antiplatelet drugs in these patients, aside from the studies merely comparing different treatment groups and reporting increased bleeding risk [18, 19]. The main goal of this study was to fill this gap and explore these issues in more detail.

We observed clear *ex vivo* haemostatic differences between a generally healthy younger population sample and our group of PP. The differences described point to increased platelet reactivity in these patients. This is particularly important as cardiovascular disease of some kind was present in virtually all these patients. In particular, the presence of type 2 diabetes mellitus, which is associated with metabolic syndrome and cardiovascular diseases, appeared to be the major disease factor for modified platelet reactivity in PP patients. Hence, these differences should be taken into account when considering antiplatelet therapy.

The first-line drug currently used in antiplatelet therapy is ASA, an inexpensive and efficient drug with relatively few side effects at the low clinically administered antiplatelet dose. However, (pseudo)resistance to ASA renders this drug less efficient or even ineffective [20, 21]. This phenomenon exhibits high prevalence in patients with metabolic disorders, further complicating their treatment [22]. Moreover, the prevalence of resistance to ASA in older patients is unknown, necessitating a comprehensive investigation in this area. In our investigation, ASA in a concentration of 30 µM was ineffective, decreasing the relative aggregation by a mere but insignificant 14%. This stands in contrast to our study with generally healthy people, where a significant decrease of 25% was documented. It should be emphasized that 30 µM is the approximate maximal concentration of ASA which might be achievable from low-dose ASA tablets [23]. Importantly, when compared to generally healthy younger persons, 70 µM of ASA was less active in our PP, decreasing the aggregation by 40%, compared to an almost 70% decrease in the control group. The same trend was observed in our prior study with type 1 diabetic patients, which also showed decreased efficacy of our tested antiplatelet drugs on the AA-triggered aggregation [13]. On the other hand, 4-MC has retained its efficacy in older patients, showing a similar decrease in both AA and collagen-triggered aggregation *ex vivo*. These observations support our hypothesis that 4-MC may present a viable antiplatelet drug in these PP. Due to its small size and simple, easily modifiable structure, 4-MC or its close derivatives might represent novel antiplatelet compounds with preserved or even better effects in older patients.

An alternative to ASA, indobufen, has shown comparable efficacy with a decreased bleeding risk in diabetic patients, offering a safer option with a similar mechanism of action [24, 25]. However, it should also be emphasized that it might be less efficient when compared to ASA in acute coronary syndrome patients, at least according to major adverse cardiovascular and cerebrovascular events analysis [26]. As 4-MC acts on the arachidonic acid-thromboxane pathway, it might also be a suitable alternative to ASA or indobufen.

If the response to ASA is inadequate, P2Y_12_ antagonists are administered, either alone or in combination with ASA. Data on clopidogrel are conflicting; it has been shown to provide better outcomes in coronary artery disease in monotherapy compared to ASA, as well as increased mortality in other studies [27, 28]. The major problem with clopidogrel is its complicated and not fully understood pharmacokinetics, as clopidogrel is inactive by itself and must be metabolized in two steps into its active metabolite. It is well known that the conversion is insufficient in some patients, as activation can be burdened by genetic polymorphism in drug-metabolizing enzymes, drug-drug interactions, and some unclear factors. In our study, we have selected ticagrelor, as it is not a prodrug and hence does not suffer from such variability as much as its predecessor. It has a very good efficacy but also a risk of side effects with bleeding being both the most common and serious. Additionally, its cost does not make it a good alternative, particularly in poorer countries. Novel drugs such as vorapaxar, a PAR1 antagonist, have been considered, but it has been pulled from both the EU and US by 2017 for commercial reasons. However, other antagonists of PAR receptors are being investigated for the prevention of thrombotic events, such as BMS – 986,141 [29–32]. We have not included the most powerful antiplatelet drugs, Gp_IIb/IIIa_ receptor antagonists, as they are indicated for acute reperfusion in a hospital setting only and have to be administered parenterally [33, 34]. It would definitely be worth investigating their effect in our group of PP, but we did not include them for two reasons: 1) the inability to add additional tests due to a relatively large volume of blood already collected, and 2) the fact that they are used in acute myocardial infarction, and such patients were not the focus of our study.

Similar to ASA, the significantly lower effect of both ticagrelor and vorapaxar in our group of PP is of note. Our data suggest that these novel antiplatelet drugs may not provide a sufficient effect in older patients when given in relevant concentrations. The concentrations of both compounds were established previously in our experiments. Vorapaxar in a concentration of 1 µM blocked about 50% of platelet aggregation in young healthy donors [35]. Similarly, the response to 500 nM of ticagrelor resulted in a similar 50% inhibition without age impact (our unpublished data). As ADP antagonists are the preferred treatment option at the moment in cases of dual antiplatelet treatment or ASA-contraindication, this is a very limiting and concerning observation. 4-MC could potentially also be used in combination with these drugs, as its molecular mechanism of action differs and hence may act additively.

The paradoxically better blood lipid parameters in the older patient group were most likely caused by hypolipidemic therapy, which was highly prevalent in PP. Aging is typically connected to both physiological and pathophysiological decreases in glomerular filtration. Renal aging presents itself in the loss of functional nephrons, decreased glomerular filtration and tubular secretion, and a loss of endocrine function [36, 37]. Creatinine data were within the reference range in both groups, but decreased kidney function in PP may have contributed to the observed differences, as creatinine correlated with inflammatory markers and they were significant predictors in our mathematical modelling. The potential impact of medication affecting kidney function should also be considered in more detail in the future, as both diuretics and inhibitors of the renin-angiotensin system are frequently administered [38]. Correlation analyses of our anthropometric and biochemical parameters have brought several interesting observations to our attention. While a positive correlation of BMI with platelet aggregation was expected (as overweight and obesity are associated with cardiometabolic disease), the negative correlations with LDL cholesterol and lipid levels were surprising in general. Studies published so far clearly demonstrate an increase in the risk of cardiovascular events and increased platelet aggregation with an increased LDL level [39, 40]. It was also shown that cholesterol depletion decreases platelet reactivity to collagen and TRAP [41, 42]. In fact, in our study, the platelet reactivity was blocked less by the higher dose of ASA in the case of lower LDL cholesterol. The differences in results observed in our study and in previous studies including ours are difficult to explain, due to the very high number of drugs and the number of diagnoses in these patients. Moreover, our mixed-linear modelling did not confirm such a relationship.

It is also nearly impossible to determine how much the platelet response was affected by the presence of a wide range of drugs in these patients. The positive effect of ACE inhibitors on metabolic parameters has already been established, as have the negative effects of diuretics and beta blockers, all of which were common in our patients [43–45]. As our patients had 10 drugs on average, with a high prevalence of anticoagulants, their influence on metabolic parameters should not be underestimated. As expected, both the exclusion of systemic glucocorticoids and micronized flavonoids modified the results. It is still unknown what fraction of these flavonoids is absorbed and how much they also yield small colonic metabolites, such as 4-MC, which can be absorbed in the large intestine. Their removal led to the loss of statistical significance of the effect of the higher concentration of ASA in both AA and collagen-induced aggregation and lowered the significance of the effect of the higher concentration of vorapaxar. This may indicate that one of the mechanisms by which they contribute to improved vessel function is through optimizing platelet function.

As sterile inflammation is often present in cardiovascular disease and leads to increased platelet reactivity and the risk of atherothrombotic events, a potent anti-inflammatory agent may provide some benefit. Inflammatory markers were also positively associated with the effect of anticoagulants in type 1 diabetic patients [46]. Inflammation of the endothelium causes decreased production of protective factors, such as nitric oxide and prostacyclin. Additionally, inflamed endothelium has decreased capacity for cleaving of von Willebrand factor through metalloproteases, leading to even more platelet recruitment. It can also be noticed that ristocetin, which activates the binding of von Willebrand factor to the platelet receptor glycoprotein Ib, caused stronger aggregation in older patients in our study when compared to healthy controls. Lastly, the recruitment of immune cells in inflammation, such as neutrophils, introduces yet another factor that aggravates the risk of thromboembolic events [47]. Our study confirmed this relationship, as inflammatory markers were predictors of platelet reactivity.

Glucocorticoids, a very potent group of drugs with anti-inflammatory activity, were hence expected to significantly affect platelet function. Their removal from our analysis led to a lower response to ristocetin, a lower effect of ASA 70 μM in collagen-induced aggregation and, at first sight paradoxically, a better effect in the percentage of inhibition by ASA 30 μM in arachidonic acid-induced aggregation. As glucocorticoids inhibit phospholipase A_2_ and hence lead to an overall lower production of prostanoids which include thromboxane A_2_, this finding seems to be logical. The clinical relevance of this finding remains to be determined, as a recent study found no impact of glucocorticoid use on antiplatelet therapy [19].

On the other hand, the impact of proton pump inhibitors (PPIs) and heparins was unexpected. There is currently no literature on the direct impact of PPIs on platelet function; however, they are being used in patients with dual antiplatelet treatment to reduce the risk of gastrointestinal bleeding [48]. Regardless, the adjustment of p-values removed the significance of the effect of PPI. Low molecular weight heparins (LMWH) are highly effective anticoagulants, which are preferred to traditional unfractionated heparin. Heparin-induced thrombocytopenia is a well-known and severe side effect mediated through platelet factor 4, with lower prevalence in LMWH. When assessed turbidimetrically, heparin from various sources had a significant effect on platelet aggregation. In platelet-rich plasma, no effect or a mild proaggregation was seen. However, with washed resuspended platelets, a significant inhibition was seen with various platelet aggregation inducers, with ristocetin being the most affected. In contrast, the calcimycin-triggered platelet aggregation was not significantly changed, suggesting an extracellular mechanism of action. This effect was neutralized by preincubation with either protamine or heparinase. Interestingly, an increase in thromboxane production with a decreased response to thromboxane A_2_ was also observed [49]. Other studies have shown that unfractionated heparin and, to a lesser extent, even LMWH have potentiated platelet aggregation induced by a wide range of inducers, while decreasing their response to collagen. Additionally, this effect was blocked by P2Y_12_ antagonists, while ASA had no effect [50]. This may explain why a significant decrease in response to U-46619 was observed in our patients with anticoagulant heparin-based therapy.

This paper has several limitations. 1) Our sample size was relatively small, but we do not expect profound changes by including more patients given the large variability in terms of diseases and drugs administered to our PP. 2) Advanced age, often coupled with severe chronic diseases, causes significant changes in pharmacokinetics; hence, the same dose of some drugs may result in unexpected plasma levels. 3) Apparent differences in several characteristics between older and generally healthy younger donors, in particular in relation to age, are hardly resolvable given the lack of fully healthy donors in this age category. It should also be emphasized that clinically the antiplatelet dose of ASA is the same in all age categories. Moreover, the effect of drugs is mostly tested in healthy controls first so this group can serve as a starting point for possible modification of the doses in older patients. Hence, practically, we do not consider these differences as a major limitation of our study. 4) Our study utilized only *ex vivo* experiments, which may not fully correspond to real biological *in vivo* conditions. 5) We used solely one or a few concentrations of the drugs, and although their selection was based on previous research, they might not be fully relevant to *in vivo* conditions. 6) Due to limitations in blood sampling, we did not test blood count and liver enzyme markers in the control group.

## Conclusions

In summary, this *ex viv*o study has shown that our group of patients aged ≥ 78 years with polypharmacy differed significantly in several key aspects of platelet function and the response to antiplatelet drugs compared to a control group composed of younger, generally healthy volunteers. The decreased response to the standard antiplatelet drugs may render them ineffective or lead to an increased risk of side effects and/or drug interactions if a higher dose is utilized to maintain their efficacy. While these findings require a more robust sample size and clinical settings to be confirmed, they may influence the way in which antiplatelet therapy is prescribed in older people. In particular, the presence of type 2 diabetes mellitus was a significant predictor of platelet reactivity. A small phenolic metabolite, 4-MC, has, in most cases, retained its efficacy or even showed improved efficacy in these patients. This observation is clinically valuable but is based solely on *ex vivo* data. When tested in more relevant *in vivo* conditions, it may lead to a suitable alternative to the aforementioned standard drug regimens.

## Supplementary Information

Supplementary Material 1.

## Data Availability

The datasets generated and/or analysed during the current study are available in the ZENODO repository (https://zenodo.org/uploads/18455546).

## Abbreviations

ADP: Adenosine diphosphate
ALT: Alanine aminotransferase
ALP: Alkaline phosphatase
ASA: Acetylsalicylic acid
DMSO: Dimethyl sulphoxide
LMWH: Low molecular weight heparins
PAF: Platelet-activating factor 16
PPIs: Proton pump inhibitors
PP: Polymorbid older patients
TRAP: Thrombin receptor activating peptide 6
U-46619: 9,11-Dideoxy-11α,9α-epoxymethanoprostaglandin F_2α_
4-MC: 4-Methylcatechol
