# Supplementary material for: Platelet reactivity in polymorbid older patients and the effect of 4-methylcatechol
Source: BMC Geriatr. 2026 Jul 17;26:1292. doi: 10.1186/s12877-026-07945-2 (PMC13659959; doi:10.1186/s12877-026-07945-2)
Supplement: Supplementary file 1 [file 12877_2026_7945_MOESM1_ESM.docx]

**Platelet reactivity in polymorbid older patients and the effect of 4-methylcatechol**

Marcel Hrubša^1^, Lukáš Konečný^1^,  Michaela Sirová^2^, Slávka Homolková^2^, [Alejandro Carazo](https://link.springer.com/article/10.1007/s10557-023-07455-y#auth-Alejandro-Carazo-Aff1)^1^, [Kateřina Matoušová](https://link.springer.com/article/10.1007/s10557-023-07455-y#auth-Kate_ina-Matou_ov_-Aff3)^3^, [Lenka Kujovská Krčmová](https://link.springer.com/article/10.1007/s10557-023-07455-y#auth-Lenka_Kujovsk_-Kr_mov_-Aff3-Aff4)^3,4^, Veronika Bernhauerová^5^, [Vladimír Blaha](https://link.springer.com/article/10.1007/s10557-023-07455-y#auth-Vladim_r-Blaha-Aff5)^2^, Alena Šmahelová^2^ and [Přemysl Mladěnka](https://link.springer.com/article/10.1007/s10557-023-07455-y#auth-P_emysl-Mlad_nka-Aff1)^1*^

^1^ Department of Pharmacology and Toxicology, Faculty of Pharmacy in Hradec Králové, Charles University, Hradec Králové, Czech Republic

^2^ 3^rd^ Department of Internal Medicine-Metabolic Care and Gerontology, University Hospital and Faculty of Medicine in Hradec Králové, Charles University, Hradec Králové, Czech Republic

^3^ Department of Clinical Biochemistry and Diagnostics, University Hospital Hradec Králové, Hradec Králové, Czech Republic

^4^ Department of Analytical Chemistry, Faculty of Pharmacy in Hradec Králové, Charles University, Hradec Králové, Czech Republic

^5^ Department of Biophysics and Physical Chemistry, Faculty of Pharmacy in Hradec Králové, Charles University, Hradec Králové, Czech Republic

Supplementary Data

14 pages

**Supplementary Table 1. Exclusion Criteria Based on Organ Dysfunction**

| System / Organ | Parameter | Exclusion criterion | Note |
| --- | --- | --- | --- |
| Renal function | Serum creatinine | > 200 µmol/L (or > 2.3 mg/dL) | or eGFR < 30 mL/min/1.73 m² |
|  | eGFR (CKD-EPI) | < 30 mL/min/1.73 m² | chronic or acute renal insufficiency |
| Liver function | Total bilirubin | > 50 µmol/L | if not due to hemolysis |
|  | ALT / AST | > 3× upper limit of normal | without other explained etiology |
|  | INR | > 1.5 (in the absence of anticoagulation) | indicative of hepatic dysfunction |
| Cardiovascular system | Heart failure | NYHA class III–IV | clinically manifest |
|  | Blood pressure | systolic < 90 mmHg | persistent hypotension |
| Respiratory system | Oxygen saturation | < 90% on room air | without oxygen supplementation |
|  | PaO₂/FiO₂ ratio | < 200 | severe respiratory insufficiency |
| Haematological system | Haemoglobin | < 90 g/L |  |
|  | Platelet count | < 50 × 10⁹/L |  |
|  | Leukocyte count | < 2.0 × 10⁹/L |  |
| Metabolic parameters | Lactate | > 4 mM | marker of tissue hypoperfusion |
| Neurological status | Glasgow Coma Scale | < 12 | in the absence of sedation |

ALT / AST – ratio of the activity of alanine aminotransferase to aspartate aminotransferase; CKD-EPI, Chronic Kidney Disease Epidemiology Collaboration; eGFR, estimated glomerular filtration rate; PaO₂/FiO₂ ratio, ratio of arterial oxygen partial pressure (PaO_2_ in mmHg) to fractional inspired oxygen

**Supplementary Table 2. Blood collection and treatment.**

| Volume | Vial | Purpose |
| --- | --- | --- |
| 20 mL (2x 10 mL) | heparinized | aggregation analysis |
| 6 mL | with a clotting accelerator | serum biochemical analysis |
| 9 mL (2x 4.5 mL) | citrated | coagulation analysis* and plasma (poly)phenol metabolite analysis |

*Coagulation analysis was performed solely for control reasons, as most polymorbid elderly patients were treated with anticoagulants.

**Supplementary Table 3. Employed inducers and drugs**.

| Inducer/drug | Final concentration |
| --- | --- |
| U-46619 | 80 nM |
| PAF | 20 nM |
| Collagen | 0.16/1 μg/mL |
| Arachidonic acid | 60/200 μM |
| Acetylsalicylic acid | 30/70 μM |
| 4-methylcatechol | 10/20/240 μM |
| Ristocetin | 4/400 μM |
| TRAP | 10 μM |
| Vorapaxar | 1/5 μM |
| Adenosine diphosphate | 5 μM |
| Ticagrelor | 500 nM |

PAF, platelet-activating factor-16; TRAP, thrombin receptor agonist peptide-6

**Supplementary Table 4. Age groups of polymorbid older patients**

| Age group | Number of patients |
| --- | --- |
| 78-80 | 16 |
| 81-85 | 20 |
| 86-90 | 5 |
| 91+ | 9 |

**Supplementary Table 5. Liver function biochemical markers in polymorbid older patients**

| Parameter | Activity (µcat/L) |
| --- | --- |
| ALT | 0.37 (0.3-0.42) |
| AST | 0.47 (0.4-0.51) |
| ALP | 1.46 (1.3-1.66) |
| GMT | 0.5 (0.35-0.93) |

ALT - alanine aminotransferase; AST - aspartate aminotransferase; ALP - alkaline phosphatase; GMT - gamma glutamyl transferase

**Supplementary Table 6. Basic blood panel data for polymorbid older patients**

| Parameter | Concentration (cells/L) | patients outside of reference range (n) |
| --- | --- | --- |
| WBC | 7.67 (6.63 - 8.57) * 10 ^9^ | 12 |
| RBC | 3.78 (3.51 - 4.13) * 10 ^12^ | 28 |
| HCT | 0.34 (0.32 - 0.36) | 43 |
| PLT | 223 (205 - 247) * 10 ^9^ | 9 |

WBC -white blood count; RBC - red blood cell count; HCT – haematocrit; PLT - platelet count

**Supplementary Table 7. Adjusted p-values for multiple pairwise comparisons; drug splits**

|  | Proton pump inhibitors | Heparins | Furosemide | Beta-blockers | Metamizole and combinations |
| --- | --- | --- | --- | --- | --- |
| Arachidonic acid 60 µM | 0.72 | 0.99 | 0.57 | 0.9 | 0.8 |
| Arachidonic acid 200 µM | 0.79 | 0.99 | 0.79 | 0.9 | 0.8 |
| Arachidonic acid 200 µM + ASA 30 µM | 0.15 | 0.99 | 0.57 | 0.9 | 0.72 |
| Arachidonic acid 200 µM + ASA 70 µM | 0.28 | 0.99 | 0.99 | 0.9 | 0.72 |
| Arachidonic acid 200 µM + 4-MC 10 µM | 0.79 | 0.99 | 0.57 | 0.9 | 0.72 |
| Collagen 0.16 µg/mL | 0.96 | 0.44 | 0.73 | 0.83 | 0.86 |
| Collagen 1 µg/mL | 0.96 | 0.44 | 0.86 | 0.65 | 0.86 |
| Collagen 1 µg/mL + ASA 70 µM | 0.96 | 0.18 | 0.73 | 0.65 | 0.86 |
| Collagen 1 µg/mL + 4-MC 20 µM | 0.96 | 0.49 | 0.73 | 0.65 | 0.86 |
| Collagen 1 µg/mL + 4-MC 70 µM | 0.96 | 0.18 | 0.73 | 0.65 | 0.86 |
| TRAP 10 µM | 0.57 | 0.7 | 0.72 | 0.76 | 0.65 |
| TRAP 10 µM + Vorapaxar 1 µM | 0.86 | 0.7 | 0.72 | 0.76 | 0.65 |
| TRAP 10 µM + Vorapaxar 5 µM | 0.86 | 0.57 | 0.72 | 0.33 | 0.65 |

**Supplementary Table 8. Adjusted p-values for multiple pairwise comparisons**

|  | p values | | |
| --- | --- | --- | --- |
|  | no ASA | no glucocorticoids | no flavonoids |
| Arachidonic acid | | | |
| Arachidonic acid 60 µM | 0.36 | 0.3 | 0.387 |
| Arachidonic acid 200 µM | 0.36 | 0.514 | 0.336 |
| Arachidonic acid 200 µM + ASA 30 µM | 0.36 | 0.384 | 0.267 |
| Arachidonic acid 200 µM + ASA 70 µM | **0.004** | **0.004** | **0.008** |
| Arachidonic acid 200 µM + 4-MC 10 µM | 0.67 | 0.63 | 0.74 |
| Arachidonic acid 200 µM + ASA 30 µM % | 0.32 | 0.107 | 0.336 |
| Arachidonic acid 200 µM + ASA 70 µM % | **0.004** | **0.004** | **0.008** |
| Arachidonic acid 200 µM + 4-MC 10 µM | 0.629 | 0.493 | 0.74 |
| Collagen | | | |
| Collagen 0.16 µg/mL | 0.35 | 0.57 | 0.4 |
| Collagen 1 µg/mL | **0.004** | **0.006** | **0.01** |
| Collagen 1 µg/mL + ASA 70 µM | 0.064 | 0.112 | 0.096 |
| Collagen 1 µg/mL + 4-MC 20 µM | **0.004** | **0.003** | **0.003** |
| Collagen 1 µg/mL + 4-MC 70 µM | **0.004** | **0.003** | **0.003** |
| Collagen 1 µg/mL + ASA 70 µM % | 0.32 | 0.373 | 0.4 |
| Collagen 1 µg/mL + 4-MC 20 µM % | **0.004** | **0.003** | **0.003** |
| Collagen 1 µg/mL + 4-MC 70 µM % | 0.32 | 0.434 | 0.147 |
| Ristocetin | | | |
| Ristocetin 4 µM | **0.005** | **0.006** | **0.002** |
| Ristocetin 400 µM | **0.005** | **0.02** | **0.002** |
| Ristocetin 400 µM + 4-MC 250 µM | **0.003** | **0.009** | **0.002** |
|  |  |  |  |
| TRAP | | | |
| TRAP 10 µM | 0.92 | 0.96 | 0.99 |
| TRAP 10 µM + Vorapaxar 1 µM | 0.92 | 0.96 | 0.99 |
| TRAP 10 µM + Vorapaxar 5 µM | **0.003** | **0.003** | **0.012** |

**Supplementary Fig. S1 Comparison of patients treated or not treated with (w) acetylsalicylic acid.** Significant differences are emphasized in red. Normality was assessed with the Shapiro-Wilk test. Pairwise comparisons were performed with unpaired t-test or the Mann-Whitney test for non-parametric distributions.

**Supplementary Fig. S2 Relative inhibition of aggregation in polymorbid older patients.** The inhibition was calculated separately for each patient using the corresponding sample with the solvent and inducer. Normality was assessed using the Shapiro-Wilk test. Statistical significance was calculated using ANOVA/Kruskal-Wallis test followed by the post hoc Dunn's/Holm-Šidák multiple comparisons test. Pairwise comparisons were performed with the Mann-Whitney test. **A**: arachidonic acid, **B**: collagen, **C**: thrombin receptor agonist peptide-16 (TRAP). ASA = acetylsalicylic acid, 4-MC = 4-methylcatechol

**Supplementary Fig. S3 Pairwise comparison of relative inhibition of aggregation in polymorbid older patients (PP, red) and healthy controls (blue).** The inhibition was calculated separately for each patient using the corresponding sample with the solvent and inducer. Normality was assessed using the Shapiro-Wilk test. Statistical significance was calculated using the Mann-Whitney test/ unpaired t-test. **A**: arachidonic acid (AA, 200 µM) after pretreament with acetylsalicylic acid 30 µM, **B**: AA (200 µM) after pretreament with acetylsalicylic acid (70 µM), **C**: AA (200 µM) after pretreament with 4-methylcatechol (10 µM), **D:** Collagen (1 µg/mL) after pretreament with acetylsalicylic acid (70 µM), **E**: Collagen (1 µg/mL) after pretreament with 4-methylcatechol (20 µM), **F**: Collagen (1 µg/mL) after pretreament with 4-methylcatechol (70 µM)

**Supplementary Fig. S4 Pairwise comparison of relative inhibition of aggregation in polymorbid older patients (PP, red) and healthy controls (blue).** The inhibition was calculated separately for each patient using the corresponding sample with the solvent and inducer. Normality was assessed by the Shapiro-Wilk test. Statistical significance was calculated using the Mann-Whitney test/ unpaired t-test. **A**: TRAP (10 µM) after pretreatment with vorapaxar (1 µM), **B**: TRAP (10 µM) pretreatment with vorapaxar (5 µM), **C**: ADP (5 µM) after pretreatment with ticagrelor (500 nM), **D**: ristocetin (400 µM) after pretreatment with 4-methylcatechol (250 µM)

**Supplementary Fig. S5 Pairwise comparison of responses to platelet-activating factor 16 (PAF) and U-46619 in polymorbid older patients (PP, red) and healthy controls (blue**). Data expressed as area under the curve (AUC). Normality was assessed using the Shapiro-Wilk test. Statistical significance was calculated using the Mann-Whitney test/ unpaired t-test. **A**: U-46619 (80 nM), **B**: PAF-16 (20 nM)

**Supplementary Fig. S6 The difference in the response to arachidonic acid after pretreatment with a lower concentration of acetylsalicylic acid (30 µM) between polymorbid older patients (PP) and generally healthy donors after removal of blood donors taking glucocorticoids.** Data are expressed as % of aggregation relative to individual responses to 200 µM of arachidonic acid. Normality was assessed using the Shapiro-Wilk test, and pairwise comparison was done by the non-parametric Mann-Whitney test

**Supplementary Fig. S7** **Relationship between age or biochemical parameters and the response to 70 µM of ASA in arachidonic acid-induced aggregation.** Simple linear regression was used for analysis. Patients treated with ASA were removed from the analysis. KYN/TRP: kynurenine:tryptophan

**Supplementary Fig. S8** **Relationship between age or serum kynurenine/tryptophan (KYN/TRP) ratio and the response to 20 µM of 4-MC in collagen-induced aggregation.** Simple linear regression was used for analysis. Patients treated with ASA were removed from the analysis

**Supplementary Fig. S9** **Relationship between age or serum kynurenine/tryptophan (KYN/TRP) ratio and the response to 500 nM of ticagrelor in ADP-induced aggregation.** Simple linear regression was used for analysis. Patients treated with ASA were removed from the analysis

**Supplementary Fig. S10** **Correlation analysis of anthropometric, biochemical, and aggregometry values in the group of polymorbid older patients**. Normality was assessed using the Shapiro- Wilk´s test. The majority of variables had a non - Gaussian distribution, therefore two-sided Spearman correlation coefficients were calculated. Only the polymorbid older patients not treated with ASA were included in the analysis
